# The Fabrication of a 3D-Printed Nerve Guidance Conduit Using Heterogeneous Composite Materials and Its Effectiveness on Sciatic Nerve Defects of a Rabbit Model

**DOI:** 10.3390/polym18010109

**Published:** 2025-12-30

**Authors:** Hyung Bae Kim, Soohyun Kwon, Yong-Hun Kim, Jin Sup Eom, Jin-Hyung Shim, Hyun Ho Han

**Affiliations:** 1 Department of Plastic and Reconstructive Surgery, Asan Medical Center, University of Ulsan College of Medicine, Songpa-gu, Seoul 05505, Republic of Korea; hyungbae6051@naver.com (H.B.K.); jinsupp@amc.seoul.kr (J.S.E.); 2 Research Institute, T&R Biofab Co., Ltd., Siheung-si 15111, Gyeonggi-do, Republic of Korea; soos1204s@naver.com (S.K.); kimyh@tnrbiofab.com (Y.-H.K.); 3 Department of Mechanical Engineering, Tech University of Korea, Siheung-si 15073, Gyeonggi-do, Republic of Korea

**Keywords:** 3D printing, nerve guidance conduit (NGC), polycaprolactone (PCL), acellular dermal matrix (ADM), peripheral nerve regeneration

## Abstract

Peripheral nerve repair remains a major clinical challenge, and novel strategies such as conduit-assisted repair have been developed to improve outcomes. In this study, we fabricated a 3D-printed nerve guidance conduit (NGC) composed of polycaprolactone (PCL), a biocompatible and biodegradable polymer, combined with acellular dermal matrix (ADM) derived from porcine dermis, in order to create a multilayered PCL–ADM NGC with both favorable mechanical properties and biological activity. Twenty rabbits were divided into four groups: a negative control group, a silicone tube repair group, an autologous nerve graft group, and a group treated with the 3D-printed PCL–ADM NGCs. Sciatic nerve regeneration was assessed at 4 and 12 weeks postoperatively using electrophysiological measurements, histological staining, and electron microscopy. The PCL–ADM NGC demonstrated comparable axonal regeneration and functional recovery to autologous grafting, and it significantly outperformed silicone tubes in terms of axonal count and maximal electrophysiological response. Histological and ultrastructural analyses further confirmed that the PCL–ADM NGC facilitated organized regeneration with dense myelinated axons and reduced degenerative changes. The fabricated NGCs exhibited excellent flexibility without compromising lumen diameter, which is critical for adapting to the physiological environment of peripheral nerves. These findings indicate that combining synthetic polymers with biologically derived matrices can enhance the regenerative microenvironment and overcome limitations of traditional synthetic conduits. In conclusion, the 3D-printed PCL–ADM NGC represents a promising alternative to both silicone tube repair and autologous nerve grafting by providing structural support and bioactivity while reducing the need for donor nerve harvesting. Further studies in larger animal models and longer follow-up periods will be required to confirm long-term efficacy and support clinical translation of this technology.

## 1. Introduction

Peripheral nerve repair is a complex and delicate process, and optimizing the technical aspects of nerve coaptation is critical for achieving functional recovery. Although direct end-to-end suture repair remains the most commonly applied technique, its efficacy is limited in long-gap defects. To overcome these challenges, various artificial nerve guidance conduits (NGCs) have been developed to provide structural support and a permissive microenvironment for axonal regeneration.

Connector-assisted nerve repair was introduced to address limitations such as fascicular mismatch, suture irritation, and axonal escape [1]. However, there is still no consensus regarding the ideal connector material [2,3,4]. Silicone-based conduits, once widely used, exhibit significant drawbacks, including non-biodegradability, chronic inflammatory responses, and poor tissue integration. As an alternative, bioresorbable polymers such as polycaprolactone (PCL) have attracted attention because of their excellent mechanical strength, tunable degradation rate, and biocompatibility [5,6]. Nevertheless, synthetic materials alone often lack sufficient bioactivity to support robust nerve regeneration, particularly in long-gap injuries.

To address these limitations, recent advances in tissue engineering have explored hybrid strategies combining synthetic polymers with extracellular matrix (ECM)-derived biomaterials such as collagen to create more biomimetic environments [7]. Among fabrication techniques, electrospinning has been widely used to produce nanofibrous scaffolds composed of PCL and ECM components that mimic native endoneurial microstructures [8,9]. However, electrospinning processes typically require organic solvents such as hexafluoroisopropanol (HFIP) or chloroform, which may leave cytotoxic residues even after post-processing, thereby impairing cell adhesion and viability [10]. Moreover, electrospun scaffolds often exhibit insufficient mechanical strength and structural stability for nerve repair applications, and their random fiber orientation limits precise control over three-dimensional geometry and conduit porosity.

Conversely, coating strategies in which ECM materials are layered onto pre-fabricated PCL scaffolds have been attempted to enhance biological activity [11,12]. Yet, these surface-coating approaches frequently result in uneven ECM distribution, weak interfacial bonding, and oversized pores that hinder uniform cell adhesion and infiltration. Therefore, there remains an unmet need for fabrication strategies that combine the mechanical robustness of synthetic frameworks with the bioactivity of ECM-derived materials in a structurally controlled, solvent-free manner.

In this context, three-dimensional (3D) printing has emerged as a promising platform to overcome the limitations of electrospinning and coating techniques by enabling precise control of conduit geometry, tunable porosity, and reproducible multilayer assembly [13]. In the present study, we developed a novel multilayered NGC using a solvent-free 3D printing approach. A cylindrical PCL mesh framework was fabricated to provide mechanical strength and flexibility, followed by lamination of a porcine acellular dermal matrix (ADM) sheet onto the PCL scaffold. The ADM sheet was prepared by mechanically homogenizing the ADM in distilled water to obtain a non-uniform aqueous suspension, which was subsequently dehydrated to form a sponge-like sheet structure. An additional outer PCL layer was then printed over the ADM sheet, creating a composite PCL–ADM conduit that integrates structural integrity with biological functionality. This experimental study was designed to evaluate the efficacy and utility of this multilayered NGC compared with conventional repair methods, including silicone tubing and autologous nerve grafting.

## 2. Materials and Methods

### 2.1. Materials

The nerve guidance conduit (NGC) is designed as a three-layered, three-dimensional tubular structure composed of both synthetic and natural polymers. The innermost and outermost layers are fabricated using medical-grade PCL (Mw = 69,200; Evonik Industries AG, Darmstadt, Germany) via a 3D printing technique. The material sandwiched between these two PCL layers is a natural polymer component composed of crosslinked ADM derived from porcine dermis. The ADM layer was initially fabricated in sheet form and subsequently integrated into the conduit structure.

### 2.2. Fabrication of the Three-Layered NGC

The NGC was engineered with a three-layered tubular structure comprising an inner synthetic layer, a middle bioactive layer, and an outer mechanical support layer. The inner layer was fabricated using PCL via a 3D printing process (3DX Printer, T&R Biofab Co., Ltd., Siheung-Si, Republic of Korea), wherein PCL was heated to 120 °C and extruded through a precision nozzle to form a hollow conduit, serving as the structural backbone and substrate for subsequent layer assembly. The middle layer consisted of crosslinked ADM designed to provide a bioactive and biocompatible environment conducive to peripheral nerve regeneration. Fresh porcine dermal tissues, obtained from a certified supplier, were processed through a standardized decellularization protocol, mechanically minced in phosphate-buffered saline (PBS) into a uniform slurry, and mixed with a crosslinking solution. The slurry was cast into molds, partially dehydrated under vacuum, and mechanically pressed into flat sheets. These ADM sheets were concentrically wrapped around the pre-fabricated PCL inner conduit and freeze-dried to preserve their porous architecture and flexibility. Subsequently, the outer PCL layer was directly printed onto the ADM-wrapped construct using the same 3D printing platform, with printing parameters optimized to ensure intimate adhesion between the outer layer and the underlying bioactive sheet, thereby forming a fully integrated three-dimensional construct. Following fabrication, the assembled NGCs were thoroughly rinsed with ultra-pure water, subjected to a final freeze-drying cycle to ensure complete moisture removal, and sterilized using electron beam (E-beam) irradiation to guarantee sterility and biocompatibility of the final medical device.

### 2.3. In Vitro Cytotoxicity Test

The device samples were prepared according to ISO 10993-5 and ISO-10993-12 guidelines [14,15]. Briefly, NGCs were incubated in the extraction medium at a ratio of 0.1 g/mL. The extraction medium consisted of MEM supplemented with 10% fetal bovine serum (FBS, Gibco, Thermo Fisher Scientific, Waltham, MA, USA) and 1% penicillin-streptomycin at 37 °C, 5% CO_2_ for 48 h. Positive control material (Polyurethane film containing 0.1% zinc diethyldithiocarbamate) and negative control material (high-density polyethylene film) were extracted using identical conditions. In vitro cytotoxicity experiments were performed three times per experimental group.

### 2.4. Tensile Strength Test

Test specimens were used by whole NGC samples; five specimens were prepared. Specimens were tested in hydrated states to simulate conditions during surgery. For hydrated testing, specimens were immersed in phosphate-buffered saline (PBS, pH 7.4) at room temperature (23 ± 2 °C) for 10 min prior to testing. All measurements were performed at room temperature and 50 ± 5% relative humidity after conditioning specimens for 48 h in this environment.

Tensile testing was conducted using a universal testing machine (Instron 3433, Instron Corporation, Norwood, MA, USA) equipped with a 500 N load cell. Specimens were mounted in pneumatic side-action grips with serrated jaw faces (grip pressure: 0.2 MPa) to prevent slippage while minimizing specimen damage. The initial grip-to-grip separation (gauge length) was set at 10 mm for all tests. Tests were performed at a constant crosshead speed of 5 mm/min until specimen failure occurred. During testing, force and displacement data were continuously recorded.

### 2.5. Suture Retention Strength Test

Test specimens were used by whole NGC samples; five specimens were prepared. Specimens were tested in hydrated states to simulate conditions during surgery. For hydrated testing, specimens were immersed in phosphate-buffered saline at room temperature for 10 min prior to testing. All measurements were performed at room temperature and 50 ± 5% relative humidity after conditioning specimens for 48 h in this environment.

A 2-0 non-absorbable nylon surgical suture (NB243, AILEE Co., Ltd., Busan, Republic of Korea) was used for all tests to standardize suture diameter and mechanical properties. The suture passed through the specimen at 2.0 mm from the cut edge, perpendicular to the longitudinal axis of the specimen, forming a loop through one wall of the material.

The suture retention test was performed using the same universal testing machine employed for tensile testing, equipped with fixation grips. The lower end of the specimen was secured between clamps in the bottom jaw, while the suture loop was secured between clamps in the upper jaw. The vertical distance between the specimen clamping point and the suture insertion point was maintained at approximately 10 mm to ensure consistent testing geometry.

The suture was pulled vertically at a constant crosshead speed of 200 mm/min until one of the following failure modes occurred: suture pull-out from the specimen material, tearing of the specimen material at the suture insertion point, fracture of the suture itself. During testing, force and displacement data were continuously recorded.

### 2.6. Animal Study Design

The study was designed in a population of 20 New Zealander rabbits. The Animals were divided into 4 groups: Group A included 3 rabbits which underwent sciatic nerve disconnection as a negative control group; Group B included 6 rabbits which underwent reconstruction of the sciatic nerve with a silicone tube; Group C included 5 rabbits which underwent reconstruction of the sciatic nerve with an autologous nerve graft; Group D included 6 rabbits which underwent reconstruction of the sciatic nerve with a NGC.

All animal experiments were approved by the Institutional Animal Care and Use Committee (IACUC) of Asan Medical Center, Seoul, Republic of Korea (Approval No. [2023-12-035]). In accordance with the 3Rs principle of reduction, negative control animals (*n* = 3) were maintained at the minimum necessary number to provide baseline comparison while minimizing animal use. Experimental group sample sizes were unequal (*n*= 5 to 6) due to perioperative anesthesia-related losses during surgery, recovery, and dressing procedures.

### 2.7. Surgical Technique

The surgeries were performed under general anesthesia. The anastomosis was performed with non-absorbable threads ethilon 10-0.

**Figure 1:**
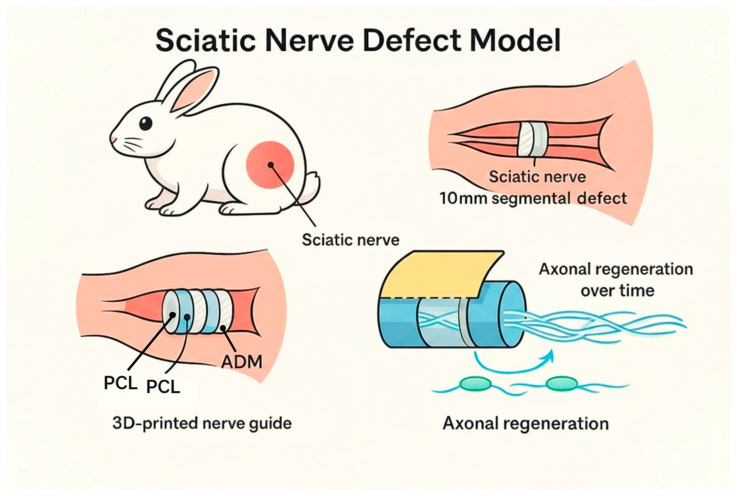
Schematic image of animal test.

The sciatic nerve was exposed through an incision in the median region of the thigh, extending from the superior edge of the trochanter to the insertion of the gluteus muscle. The sciatic nerve was simply cut in group A. The silicone tube (group B) and the nerve guidance conduit (group D) were used for direct repair of the nerve. The autologous nerve graft (group C) was harvested from the sciatic nerve (10 mm) and the nerve graft was reversed and anastomosed (Figure 1).

### 2.8. Axonal Count

Specimens were examined on a Zeiss electron microscope. Microphotographs were taken with a magnification of ×2000, and the entire cross-section was evaluated. The myelinated axons were counted using the CaseViewer (3DHISTECH Ltd., Version 2.4, Budapest, Hungary).

The number of myelinated and non-myelinated axons was evaluated 12 weeks after the operation.

### 2.9. Electrophysiological and Histological Evaluation

Electrophysiological assessments were performed at 4 and 12 weeks using electromyography. Maximal amplitude was recorded from the gastrocnemius muscle after sciatic nerve stimulation. Histological analyses included H&E and Toluidine Blue staining, and electron microscopy was performed with standard fixation and embedding protocols in vivo.

The electrophysiological evaluation was performed with the electromyography after 4 and 12 weeks. Electrical activity was recorded from the gastrocnemius muscle innervated by the tibial nerve and the distal branch of the sciatic nerve. The evoked response in this muscle could reflect the innervation across the nerve coaptation.

The stimulator was a stainless-steel monopolar wire. It was directly put on the sciatic nerve (proximal to the nerve coaptation) after careful dissection. The stimulus was a rectangular shock (duration 100 ms, frequency 1 Hz). The voltage was increased from 0.39 mV to 5.1 mV. Maximal amplitudes were evaluated. The minimal threshold was obtained when the muscle contracture and signal were observed. The recording electrodes were two monopolar stainless-steel needles inserted near the muscular mass, proximal gastrocnemius muscle and distal gastrocnemius muscle. A grounded needle was also inserted in the abdomen. Serial recordings could be made on the same animal anesthetized with either. Maximal amplitudes were evaluated

For the light microscope study, downstream of the suture (5 mm) was prepared and stained with hematoxylin-eosin (H-E) and Toluidine-Blue. For the electron microscope study, ultrathin 5 mm sections were performed downstream of the suture. Three ultrathin sections (1 nm) from each specimen were prepared on single-slotted copper grids and stained with Reynold’s lead citrate and uranyl acetate.

Specimens were examined on a Zeiss electron microscope. Microphotographs were taken with a magnification of ×400 and serial photographs of the entire cross-section of three samples were prepared.

The specimens were prepared using a standard protocol involving primary fixation in 2.5% glutaraldehyde in phosphate buffer (PB) to preserve cellular structures, followed by washing in PB. Post-fixation and staining were performed with 1% osmium tetroxide in PB to enhance electron density, especially in lipid membranes. After washing with distilled water, the samples were dehydrated through a graded ethanol series and acetone. They were then infiltrated with Epon 812 resin in increasing concentrations and left in 100% resin overnight. Finally, the samples were embedded in fresh Epon 812 resin for 6 h and polymerized in a 60 °C oven, ensuring optimal preservation and contrast for TEM imaging (HT7800, Hitachi, Tokyo, Japan).

### 2.10. Statistical Analysis

All data were expressed as mean ± SD. Statistical significance between the two groups was obtained by Student’s *t*-test. The mean number of myelinated and non-myelinated axons was considered. Values < 0.05 were considered statistically significant.

## 3. Results

### 3.1. Fabrication of 3D-Printed PCL–ADM NGC

**Figure 2:**
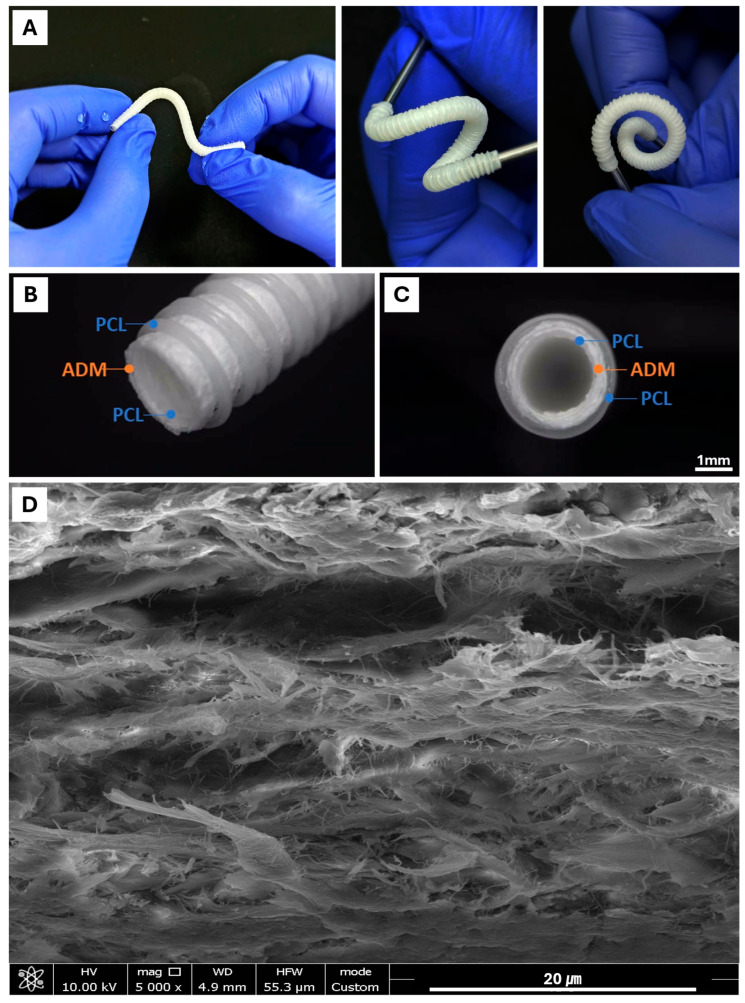
Image of 3D-printed PCL-ADM nerve guidance conduit ((**A**): flexible characteristic of the connector, (**B**): Isometric view, (**C**): Cross-sectional view, (**D**): SEM image of the fibrous ADM sheet part).

As shown in Figure 2A, we successfully fabricated a multilayered cylindrical NGC using a 3D printing process. The structure consisted of three distinct layers, with the outer and inner layers composed of PCL, providing mechanical stability and structural integrity. An ADM sheet was integrated between the PCL layers to enhance bioactivity and cellular compatibility. The NGC exhibited excellent flexibility without any significant reduction in the inner lumen diameter, as demonstrated by its ability to bend freely while maintaining its cylindrical shape (Figure 2A). Figure 2B,C confirm the tight adhesion between the PCL layers and the ADM sheet, with no visible delamination at the interface. Scanning electron microscopy (SEM) of the ADM region (Figure 2D) revealed a preserved fibrous ECM architecture favorable for cellular attachment and axonal growth.

### 3.2. In Vitro Cytotoxicity Evaluation with Mouse Fibroblast

**Figure 3:**
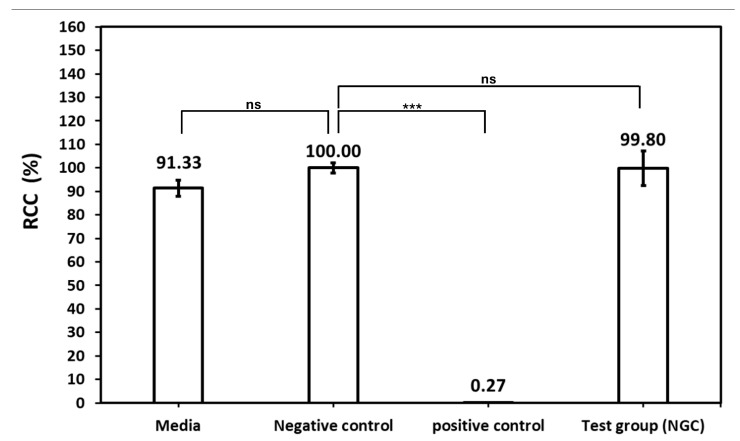
RCC result of cytotoxicity test (significance levels: *** *p* < 0.001; ns, not significant).

The biocompatibility of NGC was evaluated through in vitro cytotoxicity testing using mouse fibroblasts. The results of the live/dead viability assay are presented in Figure 3 and summarized in Table 1.

Mouse fibroblast exposed to 48 h incubation with the test extract showed 99.80% cell viability (Figure 3), compared to 100% viability in the negative control. The positive control resulted in significantly reduced cell viability of 0.27%, confirming the assay’s sensitivity to detect cytotoxic responses. The test extract demonstrated cell viability values comparable to the negative control, indicating acceptable biocompatibility of the NGCs.

### 3.3. Mechanical Properties of NGC

The mechanical properties of hydrated NGC scaffolds are summarized in Table 2. The ultimate tensile strength was 4.49 ± 0.78 N, and the ultimate suture retention strength was 4.13 ± 1.40 N. All suture retention strength test specimens failed by Type I mechanism (suture pull-out from the specimen material).

### 3.4. Microscopic Findings

**Figure 4:**
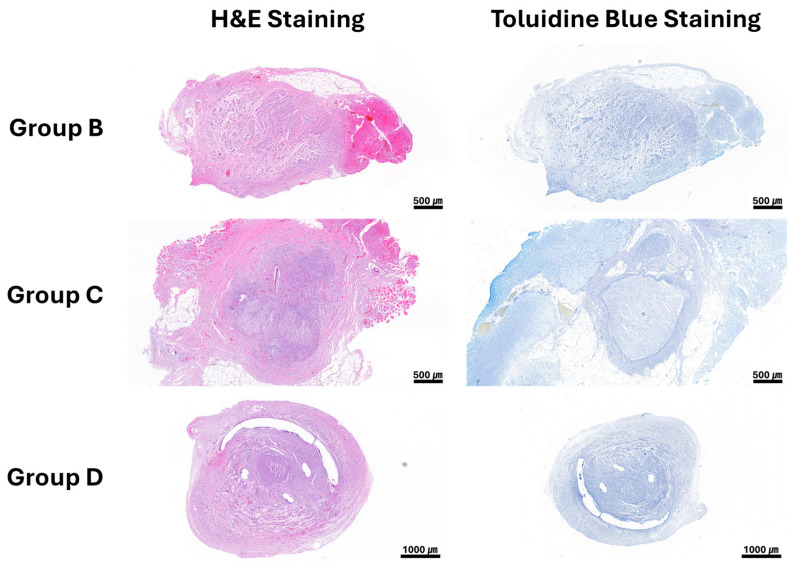
Histological images of nerve harvested at 12 weeks.

All rabbits survived without severe infection or neuroma formation at the coaptation site. In Groups B, C, and D, regenerated nervous tissue was observed along the coaptation site after 12 weeks, with no severe inflammation or fibrosis (Figure 4).

### 3.5. Result of Axonal Count

The number of myelinated and non-myelinated axons was evaluated at 12 weeks post-operation. The mean ± SD was 1564 ± 293 in Group B, 1947 ± 215 in Group C, and 1914 ± 33 in Group D (Table 3).

### 3.6. Electrophysiological Examination

Functional data of maximal amplitude after 4 and 12 weeks.

**Figure 5:**
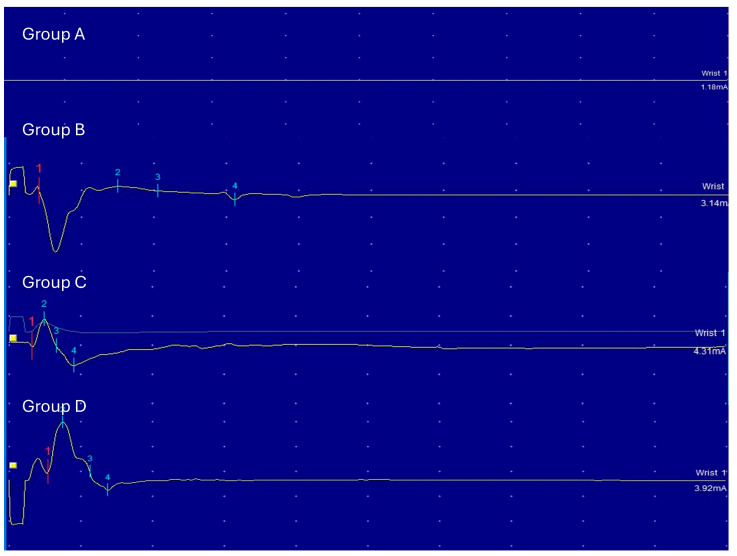
Representative examples of electromyography (EMG) waveforms demonstrating maximal amplitude. The yellow square indicates the onset of electrical stimulation. Colored numbers (1–4) represent reference markers automatically generated by the EMG device software to indicate waveform landmarks.

**Figure 6:**
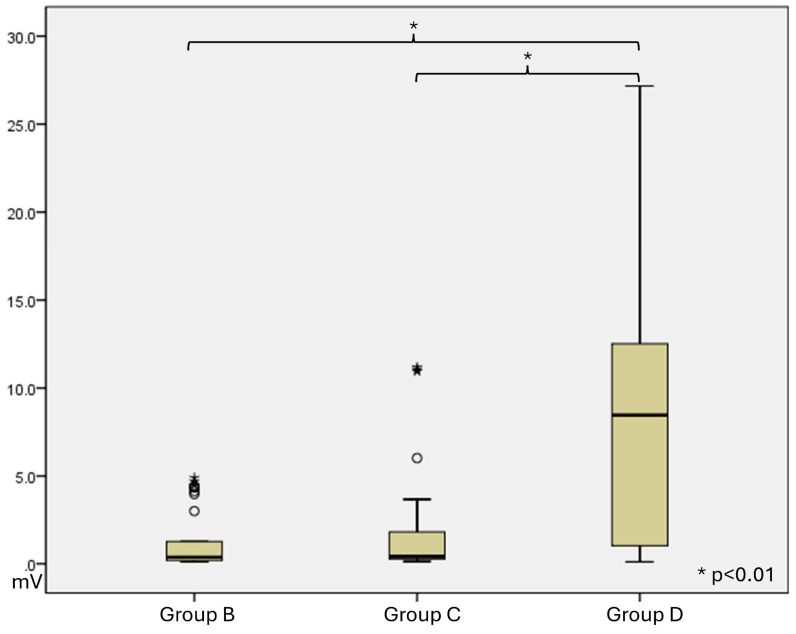
Functional data of maximal amplitude at 4 weeks after surgery. Bars represent the mean maximal amplitude of each group, and circles indicate individual animal measurements.

At 4 weeks, in group A, there was no muscle contraction. Group B, Group C, and Group D have a muscle contracture, and maximal amplitude was measured. (Figure 5) The mean ± SD of Maximal amplitude was 1.38 ± 1.70 in Group B, 1.88 ± 3.01 in Group C, and 8.79 ± 7.54 in Group D. (Table 4 and Figure 6).

**Figure 7:**
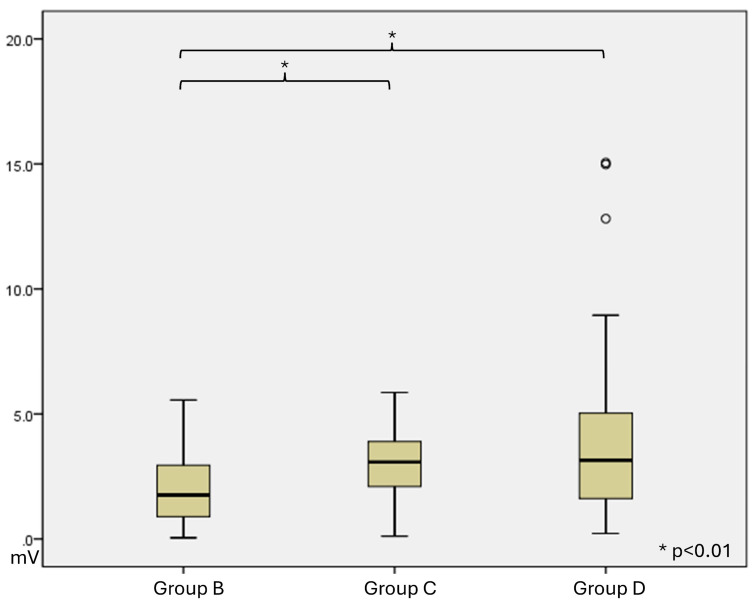
Functional data of maximal amplitude at 12 weeks after surgery. Bars represent the mean maximal amplitude of each group, and circles indicate individual animal measurements.

At 12 weeks, in group A, there was no muscle contraction. Group B, Group C, and Group D have a muscle contracture, and maximal amplitude was measured. The mean ± SD of Maximal amplitude was 2.21 ± 1.73 in Group B, 2.92 ± 1.59 in Group C, and 4.46 ± 3.91 in Group D (Table 5 and Figure 7).

### 3.7. Electron Microscopic Examination

Morphological analysis of the microphotographs was evaluated after 90 days from the operation.

**Figure 8:**
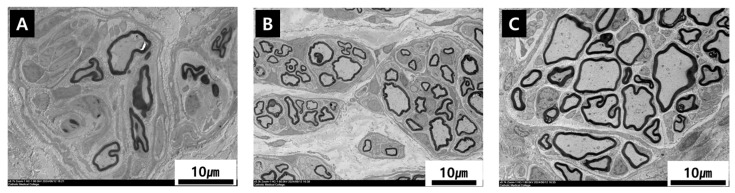
Electron microscopy image after 12 weeks ((**A**) group B, (**B**) group C, (**C**) group D).

In Group B, after 12 weeks, a few axons covered by myelin sheath and signs of Wallerian degeneration were noted. But in Groups C and D, regenerated myelinated axons and Schwann cells were densely packed, whereas in Group B, the axons had a smaller diameter. In group C and group D, thin myelinated axons were arranged in “small bundles” or “microfascicules”; they showed a parallel orientation, indicating a more systematic pattern of axonal growth (Figure 8). 

## 4. Discussion

In this study, we investigated the efficacy of a novel 3D-printed NGC made from porcine acellular dermal matrices and polycaprolactone (PCL) in repairing sciatic nerve injuries in rabbits.

In order to fabricate the cylindrical mesh-type nerve guide connector, we modified the 3D printing system by installing a rotating rod. During printing, the nozzle was positioned above the rotating rod, which allowed continuous deposition of PCL onto the turning mandrel, forming the overall tubular scaffold geometry. The utilization of PCL—a 3D-printable biomaterial recognized for its excellent elasticity—enabled the final implant to exhibit remarkable flexibility [16]. Bending tests (Figure 2A) confirmed that the conduit can be freely manipulated without compromising its lumen geometry.

Moreover, the deposited printing pattern significantly influenced the flexibility of the structure. A cylindrical architecture composed of equidistant closed-loop rings interconnected by helically oriented struts or filaments along the circumferential axis of the cylinder. The PCL-ADM NGC achieved both high deformability and sufficient mechanical strength, resisting rupture while enabling free bending. This hybrid printing strategy is a distinctive advantage achievable through 3D printing.

To enhance the biological functionality of the 3D-printed PCL structure, we incorporated an acellular dermal matrix (ADM) derived from porcine dermis. ADM is a chemically decellularized extracellular matrix that preserves native collagen and structural proteins; however, its dense internal architecture after decellularization presents a significant barrier to cellular infiltration and integration [17]. Consequently, the use of intact ADM restricts cell accessibility to the inner layers of the nerve guide conduit due to insufficient porosity.

To overcome this limitation, we applied secondary physical and chemical processing methods. Decellularized ADM were first finely shredded and suspended in water, followed by milling to produce a homogeneous fibrous slurry. This process disrupted the dense laminar organization of the ADM and yielded loosened ECM fibers. The slurry was subsequently dehydrated, compressed, crosslinked, and freeze-dried to form a fibrous sheet with enhanced porosity and improved surface accessibility.

This processed ADM sheet was wrapped around and adhered to the inner surface of the 3D-printed PCL tubular mesh, after which an additional outer PCL layer was deposited to fabricate the final three-layered nerve guide conduit. Scanning electron microscopy (SEM) revealed that the processed ADM sheet exhibited a highly porous, fibrous architecture that is favorable for cellular attachment and migration into the scaffold (Figure 2D). This optimized ECM structure is expected to facilitate axonal regeneration by enhancing cell–scaffold interactions and promoting integration within the regenerating nerve environment.

The animal study was designed in accordance with the 3Rs principle. Negative control group size was minimized (*n* = 3) to the extent compatible with statistical validity, while negative control animals still received identical surgical procedures and post-operative care as experimental groups, differing only in the absence of NGC scaffold implantation. Although negative control specimens could not be harvested for neural tissue analysis due to the intentional absence of nerve transaction and bridging in these animals.

Our in vivo results demonstrated that the PCL-ADM NGC showed promising outcomes comparable to those achieved with conventional approaches such as silicone tube repair and autologous nerve grafting. Electrophysiological and histological evaluations specifically indicated that the conduit supported enhanced axonal regeneration and functional recovery. Collectively, these findings suggest that the PCL–ADM NGC may serve as a viable alternative to current nerve repair strategies.

The axonal count analysis revealed that Group D (NGC) exhibited numbers of myelinated and non-myelinated axons comparable to those observed in Group C (autologous graft), with both groups outperforming the silicone tube in Group B. These findings underscore the NGC’s capacity to support axonal regeneration while mitigating risks such as neuroma formation and fascicular mismatch. The comparable axon counts between Groups C and D suggest that the PCL-ADM NGC may provide reconstruction efficacy approaching that of autologous grafting, but without the need for donor nerve harvest, thereby reducing donor site morbidity.

Histological and electron microscopy analyses also provided important insights. While Group B exhibited signs of Wallerian degeneration and fewer axons with smaller diameters, Groups C and D showed densely packed myelinated axons and a more organized pattern of regrowth. The NGC in Group D facilitated a similar microfascicular structure as seen in the autograft group, which is essential for effective nerve regeneration and functional recovery. The ability to recreate this organized architecture without the need for nerve harvesting represents a major advantage.

The concept of nerve coaptation using a nerve guidance conduit (enturbulation) is not new. Several clinical studies have demonstrated the benefits of connector-assisted coaptation over traditional suture repair [2,4,18,19,20,21,22]. Various nerve guidance conduit materials are currently available, each with distinct advantages and limitations. Among them, the porcine small intestinal submucosa-derived nerve guidance conduit (Axoguard Nerve Connector; AxoGen, Alachua, FL, USA) has recently gained popularity as a material of choice for this application [23]. This material undergoes rapid revascularization and remodeling, ultimately forming a permanent, epineurium-like protective layer [24]. A cross-linked bovine collagen-based implant (NeuraGen NGC; Integra LifeSciences, Plainsboro, NJ, USA) is an artificial, bioabsorbable hollow conduit composed of purified bovine type I collagen. It has demonstrated effective nerve regeneration in small-diameter, short (<3 cm) sensory nerve defects, but evidence for its use in larger sensory or motor nerve defects remains limited and inconsistent [19,25]. A collagen-coated polyglycolic acid (PGA) nerve conduit (Nerbridge; Toyobo Co., Ltd., Osaka, Japan) has also emerged as a promising nerve-end capping device for the treatment of painful amputation neuromas [26]. Additionally, PCL/PLA blend conduit has also been introduced as a nerve conduit [27].

Nerve guidance conduits are primarily designed for two key purposes: reconstructing short-gap nerve defects and achieving physiologic coaptation during nerve repair. Evidence suggests that nerve guidance conduits are effective for nerve defects measuring less than 1–5 mm, providing a scaffold that supports axonal regeneration without requiring donor nerves [1]. Comparative studies have shown that, relative to hand-sewn sutures, nerve guidance conduits yield superior outcomes in cases of clean nerve transections without defects [2,3,4,20]. This advantage is attributed to their ability to reduce tension at the repair site, prevent axonal escape, and minimize complications such as inflammation and fibrosis, ultimately promoting improved functional recovery.

Autologous nerve grafting remains a well-established option for peripheral nerve repair; however, it is associated with significant drawbacks [28]. Harvesting a donor nerve inevitably results in donor site morbidity, including sensory deficits and potential neuroma formation. Moreover, the limited availability of donor nerves restricts its application, particularly in extensive nerve defects. The harvesting procedure also prolongs operative time and increases the risk of complications, such as infection or scarring. Allogeneic nerve grafts (allonerves) represent a potential alternative, as they eliminate the need for donor site harvesting. However, their widespread clinical adoption is hindered by high cost and limited accessibility. Allonerves require extensive processing to remove immunogenic components and ensure biocompatibility, contributing to their steep price. In addition, regulatory restrictions and logistical challenges in sourcing and storage further limit their availability in many regions.

The limitations of this study include its relatively small sample size, which may reduce the statistical power and generalizability. In addition, the use of a rabbit model, while informative, may not fully replicate the complexity of human peripheral nerve injuries and repair processes. The follow-up period of 12 weeks, though sufficient for comparative evaluation of the nerve-regenerative performance between the developed 3D-printed NGC and the control groups, remains limited for assessing complete and long-term functional nerve regeneration. A longer observation period would be necessary to fully capture outcomes such as sustained functional recovery, long-distance axonal maturation, and potential delayed complications. Furthermore, although the 3D-printed NGC demonstrated promising results, direct comparisons with a broader range of commercially available nerve repair devices were not included, limiting the scope of the study. Future investigations employing larger animal models, extended follow-up durations, and more comprehensive comparisons across nerve repair modalities will be essential to validate these findings and support clinical translation.

## 5. Conclusions

In conclusion, 3D-printed PCL-ADM NGC assisted nerve coaptation. PCL-ADM NGC offers a promising alternative to both silicone tube repair and autologous nerve grafting. By providing a scaffold that supports axonal regrowth and reduces complications, the PCL-ADM NGC may improve functional outcomes in nerve repair surgeries. Further studies with larger animal models and longer follow-up periods will be essential to confirm these findings and assess the long-term efficacy of this technique in clinical settings.

## Figures and Tables

**Table 1:** Cytotoxicity test results.

Culture Condition	Total Cell (10^5^ Cells/mL)	Live Cell (10^5^ Cells/mL)	RCC (%)
Media	15.50	15.27	91.33
Negative control	17.03	16.53	100
Positive control	0.74	0.05	0.27
Test group (NGC)	17.03	16.50	99.80

**Table 2:** Ultimate tensile strength and suture retention strength.

Test	Ultimate Strength (N)	Number of Test Specimens
Tensile	4.49 ± 0.78	5
Suture	4.13 ± 1.40	5

**Table 3:** Axon counts.

	Axon Counts
Group B; silicone	1564 ± 293
Group C; autograft	1947 ± 215
Group D; NGC	1914 ± 333

**Table 4:** Electrophysiologic data of threshold and maximum amplitude at 4 weeks.

	Minimum Stimulation Threshold (mA)	Maximum Amplitude at Minimum Threshold (mV)	Maximum Amplitude at Maximum Threshold (mV)
Group B; silicone	1.66 ± 1.08	0.71 ± 1.04	0.98 ± 1.62
Group C; autograft	1.22 ± 0.85	0.73 ± 0.73	2.86 ± 3.89
Group D; NGC	0.78 ± 0.55	3.14 ± 3.81	11.30 ± 14.49

**Table 5:** Electrophysiologic data of threshold and maximum amplitude at 12 weeks.

	Minimum Stimulation Threshold (mA)	Maximum Amplitude at Minimum Threshold (mV)	Maximum Amplitude at Maximum Threshold (mV)
Group B; silicone	0.93 ± 0.81	1.43 ± 0.43	2.05 ± 1.95
Group C; autograft	0.39 ± 0.00	2.39 ± 2.05	4.15 ± 2.98
Group D; NGC	0.58 ± 0.32	2.59 ± 3.12	4.66 ± 4.36

## Data Availability

The datasets generated and analyzed in the present study are available from the corresponding authors upon reasonable request.

## References

[1] Ducic I., Safa B., DeVinney E. (2017). Refinements of nerve repair with connector-assisted coaptation. Microsurgery.

[2] Aberg M., Ljungberg C., Edin E., Millqvist H., Nordh E., Theorin A., Terenghi G., Wiberg M. (2009). Clinical evaluation of a resorbable wrap-around implant as an alternative to nerve repair: A prospective, assessor-blinded, randomised clinical study of sensory, motor and functional recovery after peripheral nerve repair. J. Plast. Reconstr. Aesthetic Surg..

[3] Boeckstyns M.E., Sørensen A.I., Viñeta J.F., Rosén B., Navarro X., Archibald S.J., Valss-Solé J., Moldovan M., Krarup C. (2013). Collagen conduit versus microsurgical neurorrhaphy: 2-year follow-up of a prospective, blinded clinical and electrophysiological multicenter randomized, controlled trial. J. Hand Surg..

[4] Alligand-Perrin P., Rabarin F., Jeudy J., Césari B., Saint-Cast Y., Fouque P.A., Raimbeau G. (2011). Vein conduit associated with microsurgical suture for complete collateral digital nerve severance. Orthop. Traumatol. Surg. Res..

[5] Yao Z., Yuan W., Xu J., Tong W., Mi J., Ho P.C., Chow D.H.K., Li Y., Yao H., Li X. (2022). Magnesium-Encapsulated Injectable Hydrogel and 3D-Engineered Polycaprolactone Conduit Facilitate Peripheral Nerve Regeneration. Adv. Sci..

[6] Saremi J., Khanmohammadi M., Azami M., Ai J., Yousefi-Ahmadipour A., Ebrahimi-Barough S. (2021). Tissue-engineered nerve graft using silk-fibroin/polycaprolactone fibrous mats decorated with bioactive cerium oxide nanoparticles. J. Biomed. Mater. Res. Part A.

[7] Li T., Javed R., Ao Q. (2021). Xenogeneic Decellularized Extracellular Matrix-based Biomaterials For Peripheral Nerve Repair and Regeneration. Curr. Neuropharmacol..

[8] Yu X., Zhang T., Li Y. (2020). 3D Printing and Bioprinting Nerve Conduits for Neural Tissue Engineering. Polymers.

[9] Juarez-Navarro K.J., Guarino V., Alvarez-Perez M.A. (2025). Converging Electrospinning and 3D-Printing Technologies: From Innovative Design for Tissue Engineering to Global Patent Trends and Technology Transfer. Fibers.

[10] Zulkifli M.Z.A., Nordin D., Shaari N., Kamarudin S.K. (2023). Overview of Electrospinning for Tissue Engineering Applications. Polymers.

[11] Blum J.C., Schenck T.L., Birt A., Giunta R.E., Wiggenhauser P.S. (2021). Artificial decellularized extracellular matrix improves the regenerative capacity of adipose tissue derived stem cells on 3D printed polycaprolactone scaffolds. J. Tissue Eng..

[12] Ebrahimi Z., Irani S., Ardeshirylajimi A., Seyedjafari E. (2022). Enhanced osteogenic differentiation of stem cells by 3D printed PCL scaffolds coated with collagen and hydroxyapatite. Sci. Rep..

[13] Hu Y., Wu Y., Gou Z., Tao J., Zhang J., Liu Q., Kang T., Jiang S., Huang S., He J. (2016). 3D-engineering of Cellularized Conduits for Peripheral Nerve Regeneration. Sci. Rep..

[14] (2009). Biological Evaluation of Medical Devices—Part 5: Tests for in Vitro Cytotoxicity.

[15] (2012). Biological Evaluation of Medical Devices—Part 12: Sample Preparation and Reference Materials.

[16] Qian Y., Zhao X., Han Q., Chen W., Li H., Yuan W. (2018). An integrated multi-layer 3D-fabrication of PDA/RGD coated graphene loaded PCL nanoscaffold for peripheral nerve restoration. Nat. Commun..

[17] Boháč M., Danišovič L., Koller J., Dragúňová J., Varga I. (2018). What happens to an acellular dermal matrix after implantation in the human body? A histological and electron microscopic study. Eur. J. Histochem. EJH.

[18] Arnaout A., Fontaine C., Chantelot C. (2014). Sensory recovery after primary repair of palmar digital nerves using a Revolnerv(®) collagen conduit: A prospective series of 27 cases. Chir. Main.

[19] Farole A., Jamal B.T. (2008). A bioabsorbable collagen nerve cuff (NeuraGen) for repair of lingual and inferior alveolar nerve injuries: A case series. J. Oral Maxillofac. Surg..

[20] Fujita S., Tojyo I., Yamada M., Go Y., Matsumoto T., Kiga N. (2014). Outcome following lingual nerve repair with vein graft cuff: A preliminary report. J. Oral Maxillofac. Surg..

[21] Lundborg G., Rosén B., Dahlin L., Holmberg J., Rosén I. (2004). Tubular repair of the median or ulnar nerve in the human forearm: A 5-year follow-up. J. Hand Surg..

[22] Lundborg G., Rosén B., Dahlin L., Danielsen N., Holmberg J. (1997). Tubular versus conventional repair of median and ulnar nerves in the human forearm: Early results from a prospective, randomized, clinical study. J. Hand Surg..

[23] Isaacs J., Safa B., Evans P.J., Greenberg J. (2016). Technical Assessment of Connector-Assisted Nerve Repair. J. Hand Surg..

[24] Nihsen E.S., Johnson C.E., Hiles M.C. (2008). Bioactivity of small intestinal submucosa and oxidized regenerated cellulose/collagen. Adv. Ski. Wound Care.

[25] Zhukauskas R., Fischer D.N., Deister C., Alsmadi N.Z., Mercer D. (2021). A Comparative Study of Porcine Small Intestine Submucosa and Cross-Linked Bovine Type I Collagen as a Nerve Conduit. J. Hand Surg. Glob. Online.

[26] Onode E., Uemura T., Hama S., Yokoi T., Okada M., Takamatsu K., Nakamura H. (2022). Nerve-End Capping Treatment with a Polyglycolic Acid Conduit for Rat Sciatic Neuroma: A Preliminary Report. J. Reconstr. Microsurg..

[27] Sun M., Kingham P.J., Reid A.J., Armstrong S.J., Terenghi G., Downes S. (2010). In vitro and in vivo testing of novel ultrathin PCL and PCL/PLA blend films as peripheral nerve conduit. J. Biomed. Mater. Res. Part A.

[28] Lans J., Eberlin K.R., Evans P.J., Mercer D., Greenberg J.A., Styron J.F. (2023). A Systematic Review and Meta-Analysis of Nerve Gap Repair: Comparative Effectiveness of Allografts, Autografts, and Conduits. Plast. Reconstr. Surg..

